# Association of Silicosis and Dermatomyositis: Case Report and Literature Review

**DOI:** 10.7759/cureus.19875

**Published:** 2021-11-24

**Authors:** Hani Chanbour, Ahmad Jiblawi, Ahmad Aboudalle, Obada Alalman, Zahia Chahine Elsett

**Affiliations:** 1 Neurosurgery, Lebanese University Faculty of Medicine, Beirut, LBN; 2 Radiology, American University of Beirut Medical Center, Beirut, LBN; 3 Anesthesiology and Critical Care, Lebanese University Faculty of Medicine, Beirut, LBN; 4 Orthopedic Surgery, Lebanese University Faculty of Medicine, Beirut, LBN; 5 Pulmonology and Critical Care, Lebanese University Faculty of Medicine, Beirut, LBN

**Keywords:** stonecutter, pulmonary involvement, autoimmune disease, dermatomyositis, silicosis

## Abstract

The association between silicosis and autoimmune diseases is not uncommon. Silicosis is well correlated with rheumatoid arthritis and systemic lupus erythematosus. However, cases of dermatomyositis associated with silicosis are relatively understudied. We report a case of a 59-year-old man with a history of cardiac, respiratory, and systemic symptoms for the duration of a year, who present to the ER with acute dyspnea, fever, chest pain, and dry cough, and was diagnosed with silicosis and dermatomyositis. In this case report, we discuss the workup done in order to reach the diagnosis, as well as the pathological mechanism that might have led to the emergence of those two entities in the same patient.

## Introduction

Silicosis is a type of pneumoconiosis, included in a broader classification of occupational lung disease. Silicosis is a result of crystalline silicon dioxide inhalation [1]. The latter is abundantly present in nature and is commonly existent in sandstone, quartz, and granite [1]. Occupations that increase the risk of silicon exposure include road workers, concrete and brick manufacturing, coal and rock mining [2].

Silicosis is reportedly associated with autoantibodies [3], including antinuclear antibodies and rheumatoid factor [4]. Caplan was the first to describe an association between rheumatoid arthritis and silica exposure in 1953 [5]. However, correlations of dermatomyositis (DM) /polymyositis (PM) with silicosis are relatively understudied and rarely reported. In the present study, we report a case of silicosis presenting with DM and cardiorespiratory symptoms; we also discuss the possible pathophysiological mechanism that led to our finding.

## Case presentation

A 59-year-old nonsmoker man, known to have hypertension, presented to the ER for acute dyspnea, tachypnea, fever of 38.5, chest pain, and dry cough. He has been working as a stonecutter for more than 30 years. His past medical history includes dry cough for more than two years, exertional dyspnea for six months prior to admission, along with myalgia and progressive proximal weakness of the lower extremities.

Physical examination revealed decreased breath sounds and fine crackles in both lung bases, multiple cutaneous dorsal eruptions on his hands with hyperkeratosis and hyperpigmentation, symmetrical proximal muscle weakness in both upper and lower extremities, and arthritis of metacarpophalangeal (MCP) joints.

**Figure 1:**
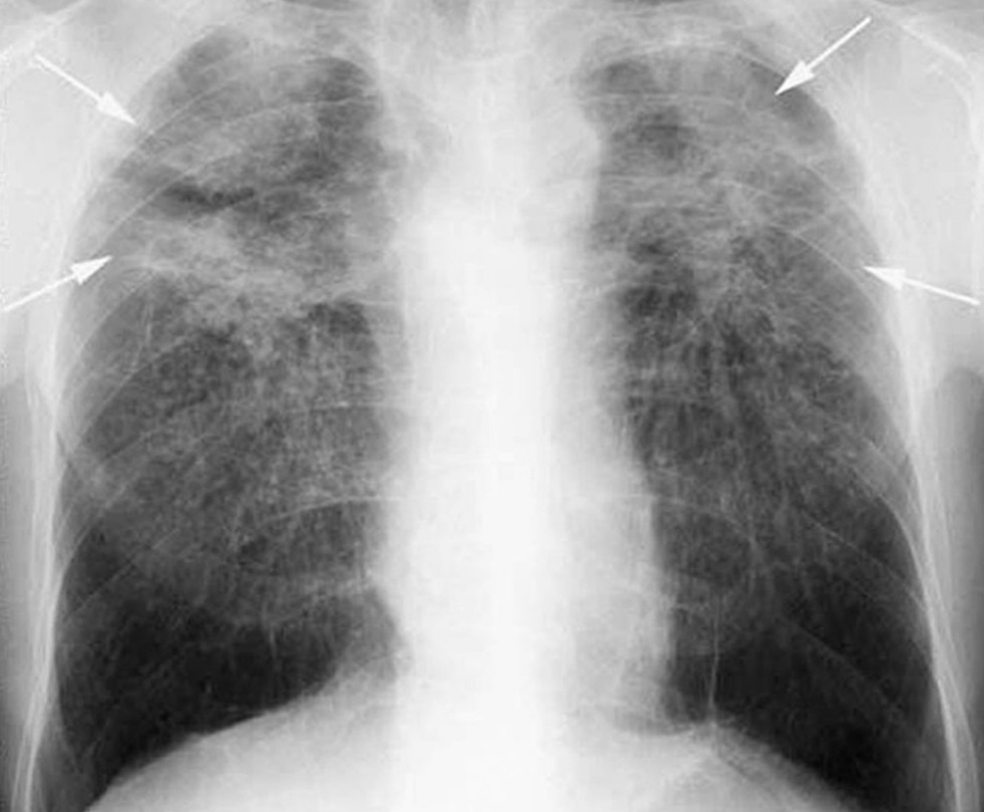
Chest x-ray upon admission: PA view, arrows showing bilateral diffuse infiltrates. Cardiomegaly is also noticed, later confirmed on CT and echocardiography. PA: posteroanterior

**Figure 2:**
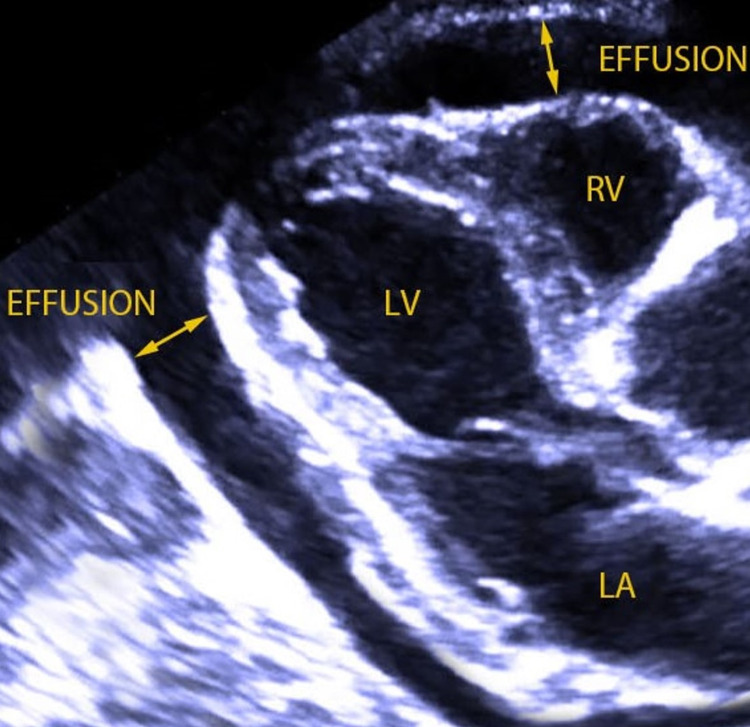
Parasternal long-axis echocardiogrpahy showing pericardial effusion, suggesting pericarditis. LV: left ventricle; LA: left atrium; RV: right ventricle

Lab results showed elevated erythrocyte sedimentation rate, C-reactive protein and creatine phosphokinase levels 103 mm/h (normal range <20 mm/h), 30 mg/L (<10 mg/L) and 250 U/L (15-190 U/L), respectively. Normal complete blood count and normal thyroid function tests were also noted. On admission, a chest x-ray showed bilateral basal infiltrates and cardiomegaly (Figure 1). Echocardiography was performed (Figure 2), suggesting a diagnosis of pericarditis for which the patient was admitted to the cardiac care unit for further monitoring and management. He was started on colchicine, steroids, and moxifloxacin.

The differential diagnosis included viral pericarditis with silicosis or an inflammatory process as advocated by his systemic symptoms, cardiac and respiratory involvement. Further questioning revealed that the patient suffered from swollen hands that improve upon functioning of the metacarpophalangeal (MCP) joints, was diagnosed with Mechanic’s hands, and was started on Methotrexate (Ebetrexat 15 mg/week and increased weekly). Additionally, he suffered from dysphonia and dysphagia to solid food usually occurring at the end of the day, because of which he lost 5 kg in weight. Those symptoms recurred at intervals of 12, six, four, and two months respectively, increasing in frequency.

**Figure 3:**
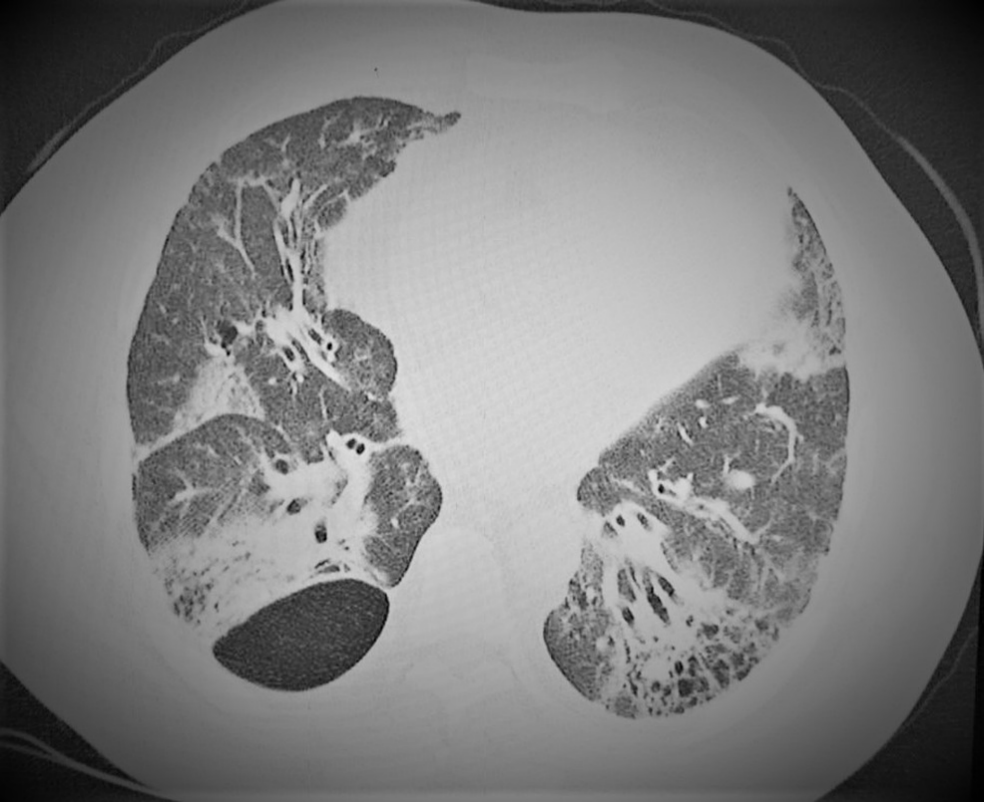
Chest CT scan: lung parenchyma window showing architectural distortion with bronchiectasis, bilateral apical and basal honeycombing pattern with diffuse perilobular septal thickening, and the presence of diffuse perilobular bilateral basal infiltrates.

**Figure 4:**
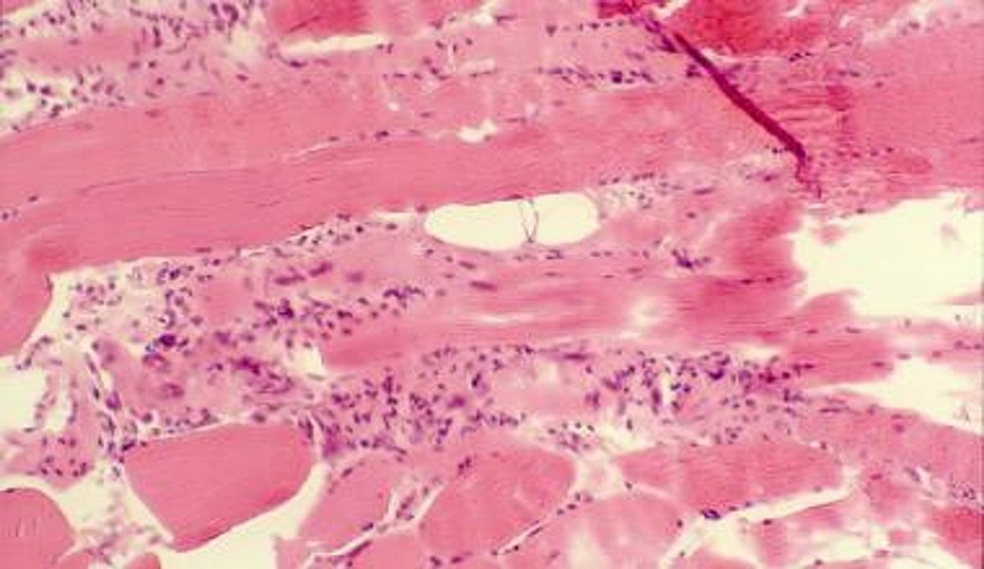
Proximal quadriceps muscle biopsy, showing features suggestive of myositis; inflammatory infiltrates of mononuclear cells in the endomysium, non-necrotic fibers surrounded and invaded by inflammatory cells.

A chest CT scan was performed and showed bronchiectasis and traction bronchiolectasis, air trapping, and fibrotic evolution with ground glass opacities (Figure 3). Pulmonary function testing showed a diffusion capacity of carbon monoxide (DLCO) of 57% of the predicted value. His labs came back showing an antinuclear antibody (ANA) level of 1/1000 with positive anti-jo-1 antibodies and rheumatoid factor (40 IU/mL). Suspicion of an acute presentation of DM with pericardial and lung involvement was supported by MRI of the thigh, performed to detect the inflammation within the said muscle group, and found evidence of myositis later confirmed on muscle biopsy (Figure 4). The latter showed inflammatory infiltrates of mononuclear cells in the endomysium, non-necrotic fibers surrounded and invaded by inflammatory cells, features consistent with myositis. Although the biopsy suggested PM at first, the lack of necrosis in muscle fibers and keeping the classification criteria of The European League Against Rheumatism (EULAR)/American College of Rheumatology (ACR) [6] in mind, a diagnosis of DM with pulmonary involvement was made. Steroids were the mainstay of treatment. Unfortunately, minimal improvement of the patient's symptoms was noted.

## Discussion

In the present study, we describe the case of a man presenting with recurrent respiratory symptoms and chest pain, associated with fever and progressive proximal muscle weakness. The patient was found to have silicosis with ground-glass opacity and honeycomb appearance of the lungs, pericardiac effusion, and DM.

The association between silicosis and autoimmune diseases is not uncommon. Silicosis was well correlated with rheumatoid arthritis with the presence of rheumatoid factor [5,7], and a series of studies highlighted the association between silicosis and systemic sclerosis [8], as well as systemic lupus erythematosus in coal miners [9]. Very few cases of DM associated with silicosis have been reported in the literature, although no direct effect has been proven until now [10]. Silicosis lung histopathology is prominently outlined as nodular, discrete lesions, which can progress to interstitial inflammation and intractable fibrosis [11]. Treatment of silicosis is mainly supportive, with no proven cure that can halt the progression of the disease [12].

DM is an autoimmune disease and a subtype of idiopathic inflammatory myopathies (IIMs), along with PM and inclusion-body myositis. It typically presents as progressive proximal muscle weakness, with or without a skin rash [13]. Some pathognomonic signs are allegedly included in the diagnosis of DM, including heliotrope rash (purple discoloration of eyelids), periorbital edema, Gottron sign (erythematous rash over extensor tendons), V-shaped rash over the face, neck, and chest, and shawl sign (rash over the back, neck, and shoulders), among others [14]. Interstitial lung disease (ILD) is one of the fearful complications of DM, it manifests as ground-glass opacities on high-resolution CT, as well as reticulations [15]. Schnabel et al. [15] found a prevalence of 32% of ILD in DM/PM, in a cohort study. Most of the patients presenting with ILD have anti-Jo1 and anti-synthetase antibodies [15]. Our patients tested positive for anti-Jo1 antibodies and had characteristics of lung involvement of both silicosis and DM.

Cardiac involvement is frequently described in DM, ranging from 9-72%. A spectrum of cardiac manifestations can be present in DM patients [16]. In fact, heart failure was the major finding, caused by necrotic and inflammatory processes of the myocardium, with decreased left ventricular ejection fraction. Furthermore, electrical abnormalities and pericardial effusion were also reported in the literature [16].

Exposure to silica initiates the production and secretion of interleukin-1 (IL-1), following the phagocytosis by the local macrophages [17]. IL-1, as a paramount mediator of inflammation, promotes a cascade of fibroblast proliferation, free radical formation, and amplification of cell-signaling pathways. Furthermore, silica directly inhibits the ability of the macrophages to defend against mycobacteria and is proven cytotoxic to alveolar type 2 cells [17].

According to Ueki et al. [18], silica induces a disorderly polyclonal proliferation of T cells, activating TcR vβ repertoires, and acting as a superantigen. This will increase the self-reactive clones of lymphocytes and cause an array of autoimmune diseases. Other pathways implying an increased vulnerability to autoimmune diseases involve elevated levels of serum-soluble Fas molecule which creates dysregulate T cells apoptosis [19], and increased autoantibodies by acting as an adjuvant on the function of B lymphocytes [3].

The National Institute of Occupational Safety and Health recommends a series of primary, secondary, and tertiary preventions. Primary prevention includes a reduction of silica exposure at the workplace, protection equipment (N-95 face masks), and supplying an adequate ventilating system. Secondary prevention suggests assessing patients at risk, and constant screening examinations (chest x-ray and biomarkers) whereas tertiary preventions focus on rehabilitation and managing complications [20].

## Conclusions

We present one of the very few studies reporting the association between silicosis and dermatomyositis. This case report highlights the correlation between silicosis and autoimmune diseases. Taken together, dermatomyositis constitutes one of the differential diagnoses of systemic diseases associated with silicosis that should be acknowledged in order to implement early treatment before the advanced progression of the disease.
